# Short-Term Effect of Daily Herbage Allowance Restriction on Pasture Condition and the Performance of Grazing Dairy Cows during Autumn

**DOI:** 10.3390/ani10010062

**Published:** 2019-12-29

**Authors:** Verónica M. Merino, Oscar A. Balocchi, M. Jordana Rivero, Rubén G. Pulido

**Affiliations:** 1 Escuela de Graduados, Facultad de Ciencias Agrarias, Universidad Austral de Chile, P.O. Box 567, Valdivia 5090000, Chile; veronicamerino@udec.cl; 2 Departamento de Producción Animal, Facultad de Agronomía, Universidad de Concepción, P.O. Box 160-C, Concepción 4030000, Chile; 3 Instituto de Producción Animal, Facultad de Ciencias Agrarias, Universidad Austral de Chile, P.O. Box 567, Valdivia 5090000, Chile; 4 Departamento de Ciencias Agropecuarias y Acuícolas, Facultad de Recursos Naturales, Universidad Católica de Temuco, Temuco 4780000, Chile; jordana.rivero-viera@rothamsted.ac.uk; 5 Rothamsted Research, North Wyke, Okehampton, Devon EX20 2SB, UK; 6 Instituto de Ciencia Animal, Facultad de Ciencias Veterinarias, Universidad Austral de Chile, P.O. Box 567, Valdivia 5090000, Chile; rpulido@uach.cl

**Keywords:** dairy cows, grazing management, milk production, grazing efficiency

## Abstract

**Simple Summary:**

Daily herbage allowance (defined as herbage mass × daily offered area) is recognized as the main grazing management factor to improve pasture utilization and milk output per hectare. Daily herbage allowances should balance the dual objectives of high milk output per hectare while maintaining the quality of the pasture to optimize the profitability of grazing-based dairy production systems. We tested two contrasting herbage allowances (17 and 25 kg dry matter (DM)/cow.day) and two levels of maize silage supplementation (4.5 and 9 kg DM/cow.day) in grazing dairy cows and measured a set of variables related to pasture management, sward characteristics, dynamic of herbage depletion throughout the grazing sessions, and cows grazing behavior, among others. Our results suggest that a more intensive grazing regime of 17 kg DM/cow per day of daily herbage allowance rather than a high level of supplementation with maize silage is appropriate to improve milk production and solids yield per hectare without affecting pasture quality or sward characteristics over the short-term.

**Abstract:**

The aim of this study was to evaluate the short-term effects of daily herbage allowance (DHA, defined as the product of pre-grazing herbage mass and offered area per animal) on pasture conditions and milk production of Holstein-Friesian dairy cows. Forty-four early lactation dairy cows were randomly assigned to one of four treatments in a 2 × 2 factorial design that tested two levels of DHA (17 and 25 kg DM/cow.day) and two levels of maize silage supplementation (MSS, 4.5 and 9 kg DM/cow.day) over a 77-day period. Low DHA decreased the post-grazing herbage mass from 1546 to 1430 kg DM/ha and the compressed sward height from 5 to 4.4 cm, while the grazing efficiency remained unaffected. Low DHA induced a faster herbage mass reduction, while the sward-height and pasture characteristics did not differ from the high DHA regime. Low DHA decreased the tiller production rates and daily lamina growth, while the leaf-production rate was not affected by the DHA. Daily increases of herbage mass were greater in the high DHA than in the low DHA treatments. Individual milk production and milk protein concentration decreased at a low DHA compared to high DHA, while the milk fat concentration was greater and the milk output per hectare increased by 1510 kg. Neither the MSS level nor the interaction DHA by the MSS level had any effect on the sward characteristics or the productivity of the cows. From these results, it is suggested that, in a high-quality pasture, using 17 kg DM/cow.day was appropriate for improving both herbage utilization and milk production per hectare while maintaining the short-term conditions of a pasture grazed by dairy cows in the autumn.

## 1. Introduction

The increased interest in pasture-based milk production systems is a consequence of lower feed cost and improved animal welfare [1]. Considering that feed costs represent approximately 80% of the total variable costs of milk production systems [2], increased profitability can be achieved through greater pasture utilization and a more efficient conversion of grass herbage into milk [3]. Moreover, pasture-based production systems reduce the competition between livestock and humans for human-edible foods [4]. Nevertheless, the success of more intensive pastoral dairy production systems requires grazing managements that ensure an adequate provision of high-quality herbage without altering sward characteristics plus a strategic supplementation with low-cost feeds that support the increased milk production and milk solids per hectare.

The daily herbage allowance (DHA), defined as the quantity of herbage (above a specified height) that is offered daily per cow [5], is widely acknowledged to be the main driver behind productivity of grazing-based dairy systems [6], depending on the pre-grazing herbage mass and stocking rate [7]. A reduction in DHA increases grazing severity and is also an option to maximize profitability per hectare (ha) of dairy systems by increasing herbage utilization (measured as the percentage of forage harvested from available pasture) and milk production per hectare [8]. Lower DHA contribute to avoiding the generation of excessive levels of refused herbage over the short-term [9].

Previous studies have focused on evaluating the effects of DHA on herbage-intake and individual milk production of dairy cows with and without energetic supplementation [6,10,11,12,13]. Most of these studies were conducted in the spring and summer or in the summer only and show that as herbage allowance increases herbage intake and milk production per cow increase, whereas efficiency of grazing decreases. Ruiz-Albarrán et al. [14] reported that reducing the DHA from 25 kg DM/day to 17 kg DM/day in winter increases milk production (+25%), milk protein (+20 kg), and milk fat (+17 kg) per hectare, without differences in individual milk performance nor the energetic and protein metabolic indicators of autumn calving dairy cows (measured as *B*-hydroxybutyrate and urea concentrations in plasma, respectively). All these studies were developed with emphasis on the animals’ requirements rather than on the pasture condition indicators, and just a few studies have also considered the manner in which the pasture responds to DHA restriction. Recently, the reduction of DHA from 30 kg DM/cow.day to 20 kg DM/cow.day on grazing management indicators, sward characteristics, dynamics of pasture depletion, and regrowth were evaluated in the spring as part of a four-years study by Merino et al. [15]. Cows grazing in low-DHA pastures decline milk production (−17.2%) and herbage intake (−2.9 kg DM/cow.day), whereas milk output per hectare increases by 27.3%, without affecting any pasture characteristics, pasture regrowth rate, or soil penetration resistance.

The amount of forage changes throughout the year, and, therefore, to optimise the profitability of pasture-based dairy systems, the level of DHA used should be selected considering the forage variation in production and quality between seasons. In the autumn, weather conditions usually decrease the pasture growth rate to 30–50 kg DM/day [16]. However, if the pasture area is sufficient, herbage can represent more than 50% of the diet of dairy cows [17]. The response to supplement is affected by herbage allowance [13], stage of lactation [18], and the genetic potential of dairy cows [9]. Thus, the level of supplementary feed must be established in accordance with the herbage offered to ensure a stable supply in quantity and quality of nutrients for milk production [19] to avoid metabolic disorders of lactating dairy cows [20] while maintaining pasture production and herbage quality over the long-term.

In order to increase our understanding of the manner in which the pasture responds to DHA restriction and how this modifies the variation of high-quality pastures throughout the season, this experiment aimed to determine the short-term effect of DHA restriction on sward characteristics, the dynamic of pasture depletion, and the pasture regrowth rate, and their subsequent effects on milk production and composition of autumn calving dairy cows supplemented with two levels of maize silage during the autumn.

## 2. Materials and Methods

### 2.1. Study Location and Treatments

The study was conducted at the Santa Rosa Experimental Station of the Universidad Austral de Chile, Valdivia, Chile (39°46′ S; 73°13′ W). The soil was a Duric Hapludand volcanic (Valdivia Series) [21] formed from volcanic ash. The local climate is classified as temperate with Mediterranean influences. The experimental period lasted 11 weeks (April to July of 2012) in addition to a 40-day pre-experimental period for evaluating the initial sward state and to adapt the animals to the experimental conditions. Weather data for the duration of the experiment were obtained from a met station located at the experimental center.

The four treatments were established in a randomized complete design with factorial arrangement of two levels of the DHA: high, 25 kg DM/cow.day and low, 17 kg DM/cow.day (measured at the ground level), and two maize silage supplementation (MSS) levels: 9 vs. 4.5 kg DM/cow.day. The levels of the DHA tested in this scenario were selected to compare two contrasting levels of DHA and prevent the generation of excessive amounts of grazing residue, according to the results of previous experiments at the same experimental site [22].

### 2.2. Pasture

**Figure 1:**
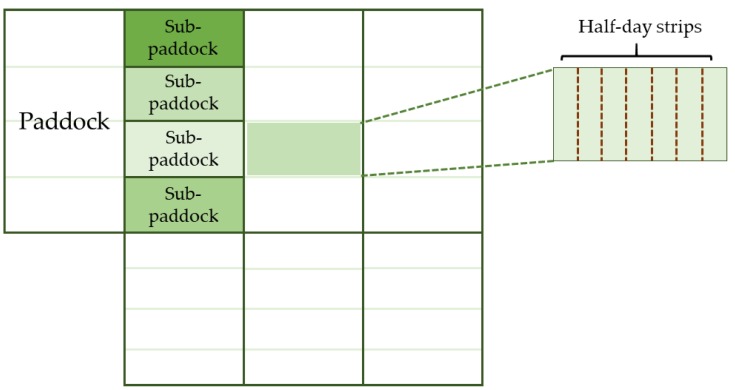
Schematic representation of the experimental area: seven paddocks, with each divided into four sub-paddocks. Each sub-paddock was divided into half-day strips. Four 11-cows herds were randomly allocated to the sub-paddocks to graze simultaneously within the same paddock.

The study utilized 10.7 hectares of two-year-old pasture that had been subjected to rotational grazing management. According to the pre-experimental measurements taken on 23 April 2012, the pasture had approximately 90% *Lolium perenne* L. and 8500 tiller/m^2^. The pasture was fertilized with 300 kg of the formulation 15-20-15 (N, P_2_O_5_, K_2_O) per hectare, which was applied at the beginning of the experimental period. Seven pasture paddocks adjacent to the milking parlor were used to facilitate the grazing measurements and management. Each of the paddocks, which ranged in size from 1.2 to 2.1 hectares, was divided into four sub-paddocks and rotationally grazed in half-day strips. The four cow herds were randomly allocated to the four sub-paddocks (Figure 1) within each paddock and grazed separately by an electric fence, according to their respective feeding treatments.

### 2.3. Animals

Forty-Four Holstein-Friesian dairy cows from the Universidad Austral de Chile’ dairy herd were split into four groups and randomly assigned to each of the four treatments. The groups were balanced according to the milk yield (24 ± 3.1 kg/cow.day), days in milk (62 ± 15.3), number of lactations (3.6 ± 1.6), body weight (538 ± 45.5 kg), and body condition score (2.98 ± 0.2 points, on a scale of 1–5) measured during the pre–experimental period. Cows were milked twice daily (am and pm) and were managed in a half-day strip grazing so that they had access to fresh pasture after each milking. In addition, all cows received 3 kg of DM of concentrate and had free access to water at all times. The concentrate provided throughout the study contained an average of 87.4% of the DM, 15.2% of crude protein (CP), 22.0% of the neutral detergent fibre (NDF), 4.5% of ash, and 13.15 MJ/kg DM of metabolizable energy (ME). The maize silage contained 36.0% of DM, 9.5% of CP, 47.2% of NDF, 4.6% of ash, 11.46 MJ/kg DM of ME, a pH of 3.9, and 5.3% of N–NH_3_. The feed samples were ground (particle size: 1 mm) and were analysed for crude protein (CP, Kjeldahl method, N × 6.25) [23], water soluble carbohydrates (WSC) [24], ash [25], and neutral detergent fibre (NDF) [26]. Metabolizable energy was estimated by regression using a “D” value (digestible organic matter/DM × 100) and was determined in vitro [27], according to Goering and Van Soest [28]. The amount of concentrate and maize silage was calculated on the base of the ME requirements for producing 24 kg milk/day [29] and were offered in two equal portions in the morning (7:00) and in the evening (15:00) at the milking-parlor.

### 2.4. Grazing Management

Paddocks were grazed when the sward reached an average pre-grazing herbage mass (HM) of 1800 to 2600 kg DM/ha, to ensure that all cows grazed in similar pasture conditions. Herbage mass and compressed sward heights (CSH) were measured using a rising plate meter (Jenquip, Feilding, New Zealand) before and after each grazing session (morning grazing from 7:00 to 15:00 and evening grazing from 16:00 to 6:00). The rising plate meter was calibrated periodically by double sampling [30], taking 10 samples pre and post-grazing (0.1 m^2^). The cutting of the sample was done at ground level with an electric mower. The samples were dried at 60 °C for 48 h, and then weighed, which transforms the result to kg DM/ha. We regressed the herbage mass (kg DM/ha) on the compressed height (½ cm) for each point, and the linear equation y = 770 + 105x was generated, where y was the herbage mass (kg DM/ha) and x was the compressed height of the sward expressed in ½ cm.

Areas provided for grazing were adjusted daily on a pre-grazing HM basis, which was estimated from 100 measurements collected with a rising plate meter, and the corresponding level of DHA assigned. The daily area was subdivided into two strips per day. The difference between pre-grazing and post-grazing HM for each grazed strip was assumed to be consumed by the cows and represented the apparent forage DM intake (DMI) per hectare. The apparent DMI per cow was estimated from the forage DMI per hectare for each grazing session (morning and evening grazing sessions) and the number of cows grazing each strip (11). The grazing efficiency was defined as the amount of forage that disappeared as a proportion of the forage offered.

### 2.5. Pasture Characteristics

Once a week, herbage samples of each strip to be grazed the next day were cut to a height of 4 cm with hand shears, and samples of ~100 g each were dried in a forced-air oven at 60 °C for 48 h to obtain the DM weight and for the assessment of the chemical composition of the herbage offered [31]. The forage chemical analysis considered the CP [23], NDF [26], digestible organic matter on a DM basis [32], and water-soluble carbohydrates [27].

Botanical composition, vertical distribution of herbage mass, and pasture species density were evaluated once during the experiment (on 11 June). Botanical composition was determined by cutting 20 herbage samples at the ground level by using a quadrat of 0.04 m^2^ randomly placed at each sub-paddock. Vertical distribution of the HM was estimated across three randomly chosen areas per sub-paddock. A stratified-clip method was used for the estimating the HM and its vertical distribution. An herbage-gripping structure holding an herbage sample was drawn from a 20 × 5 cm area while it was harvested, and it was cut into six layers at depths of 4 cm (0–4, 4–8, 8–16, 16–20, and >20 cm) from the top to the bottom of the canopy. Independent herbage samples were dried and weighed in the laboratory, and the DM herbage mass of each layer was calculated. The plant species proportions were calculated by hand-separation, and their relative contributions to the total DM value were estimated after oven drying them at 60 °C for 48 h.

The density of the pasture species was measured from eight samples per sub-paddock using the core technique developed by Mitchell and Glenday [33] and by applying the ranking method of McIntyre [34]. Within each core of 78 mm in diameter, the number of grass tillers, white clover growing points, and broadleaf species plants were counted to estimate the species density (n/m^2^) following the method developed by Bircham and Hodgson [35].

### 2.6. Herbage Depletion and Changes on Sward Morphological Components during Grazing Sessions

During three evaluation dates (24 May, 8 June, and 24 June 2012) and for 270 min of grazing sessions each day, changes in the grazed pasture conditions were determined by measuring the undisturbed sward surface height (USSH) with the first-contact technique using a sward stick [36] and by measuring the HM with a rising–plate meter [37]. Measurements were made in each strip at 30-min intervals, which enabled the grass disappearance of each grazing strip to be calculated. The reduction of the USSH and HM was defined as the difference between initial and final values for each grazing session and was expressed as a percentage of the initial value.

The grazing activity was determined by observing the 11 cows and recording the number of animals that were grazing at 10-min intervals. Cows were considered to be grazing when bowing their heads down and consuming herbage [38]. Bite rates were determined by observing the 11 cows and counting the number of bites taken over a 1-min period by using a hand-held counter, at the beginning and near the end of the grazing event. The sward morphological components (>4 cm) were measured at the beginning (T1, 8:00), middle (T2, 10:00), and end (T3, 13:00) of the grazing session. Over each evaluation date, nine herbage samples per treatment were cut 4 cm above ground level with shearing scissors and in a quadrat of 0.04 m^2^ and stored at 4 °C. Then, they were manually divided into lamina, sheath, and dead material (defined as leaves and sheaths of which more than 50% of the surface was dead) and were oven-dried at 60 °C for 48 h. Stem and inflorescence were not found during the three evaluation dates and, therefore, they were not included within the sward morphological components.

### 2.7. Pasture Regrowth Rate

After each of the three grazing sessions of herbage-depletion measurement, 10 randomly selected and recently grazed perennial ryegrass tillers (*L. perenne* L.), were chosen in each of the four strips just grazed. The selected tillers were marked with colored paper clips and monitored every three days over the regrowth period until the next grazing. The HM, lamina growth, leaf appearance, and tillering were recorded to determine the rate of perennial ryegrass regrowth. A new leaf was considered as having ‘appeared’ when its tip was visible [39]. The grass growth (kg DM/ha/day) was calculated by dividing the HM production, which was estimated through 50 CSH measurements using a rising plate meter, by the number of regrowth days, while the lamina length was measured with a graduated ruler (cm).

### 2.8. Animal Measurements

The individual milk yield was recorded electronically at daily morning and evening milking. Samples were collected weekly to determine the milk fat and milk protein concentrations by infrared spectrophotometry (MilkoScan, System 4300 Foss Electric, Hilleroed, Denmark). Cows were weighed once a week after morning milking and the body condition score (BCS) was recorded on a five-point scale [40].

### 2.9. Statistical Analyses

For the pasture-management, sward-characteristic analysis, and cows’ activity, the experimental unit corresponded to the sub-paddock, while, for the bite rate and animal performance, each cow was considered to be an experimental unit. Pasture-management data such as pre-grazing and post-grazing HM, DMI per ha and per cow, offered area and efficiency of harvesting, and pasture characteristics such as nutritional and botanical composition) were analysed by a two-way ANOVA, with the DHA level and the level of MSS used as fixed factors. Vertical distribution of HM on pre-grazing was analysed by a one-way ANOVA with the DHA as the fixed factor. The dynamics of herbage depletion and the grazing activity recorded during the three evaluation sessions were analysed with a general linear model that considered the minutes as a repeated measure. A complete randomized design with a 2 × 2 factorial arrangement was employed to study changes in the sward morphological components during the evaluation of grazing sessions, the pasture regrowth rate, the biting rate, milk yield, and milk composition. Tukey’s test method was used to compare the means when the ANOVA results were significant. All the analyses were performed with SAS (SAS Institute Inc., Cary, NC, USA) [41].

## 3. Results

### 3.1. Weather

The mean daily temperature during the experimental period was 9.6 °C, which was 0.6 °C greater than the last 10-year average. The average maximum air temperature reached 12.3 °C and an average minimum was 6.5 °C. The total rainfall during the experimental period was 410.4 mm (fallen in 36 rainy days), which was 19.2% lower over the last 10-year average.

### 3.2. Grazing Management

The pre-grazing HM and the CSH did not differ between DHA treatments, averaging 2293 kg DM/ha and 8.1 cm, respectively (Table 1). Cows grazing under low DHA conditions had 116 kg DM/ha less post-grazing HM and 0.6 cm post-grazing CSH than the high DHA regime (Table 1). However, despite the provided area being reduced by 32.4 m^2^/cow in the low DHA compared to the high DHA treatments, there was no difference in the grazing efficiencies between DHA groups. The DHA did not affect the apparent forage DMI per hectare, whereas individual forage DMI levels decreased by 2.2 kg DM under low DHA regime compared with the high DHA. Neither the level of MSS nor the interaction between DHA and the MSS had any effect on the grazing management variables.

### 3.3. Pasture Characteristics

Restricting daily herbage allowance did not affect any sward variable (Table 2). There was no difference in the vertical distribution of HM between the two DHA regimes. In both DHA groups, the greater proportion was positioned below 4 cm in height, which averaged 53.2% of the DM (Table 2).

The chemical composition of the pasture offered did not vary among the DHA treatments, which contained, on average, 12.7% DM, 24.9% CP, 11.7 MJ/kg DM of ME, and 48.6% NDF (Table 3). *L. perenne* L. was the predominant pasture species, averaging 92.6% of DM. The broadleaf species density tended (*p* = 0.0628) to increase at a low DHA. Grasses had greater density than legumes and broadleaf species, averaging 8474 tillers/m^2^. Neither the level of MSS nor the interaction between DHA and the MSS level had any effect on the sward characteristic variables.

### 3.4. Herbage Depletion and Changes in the Morphological Components during Grazing Sessions

**Figure 2:**
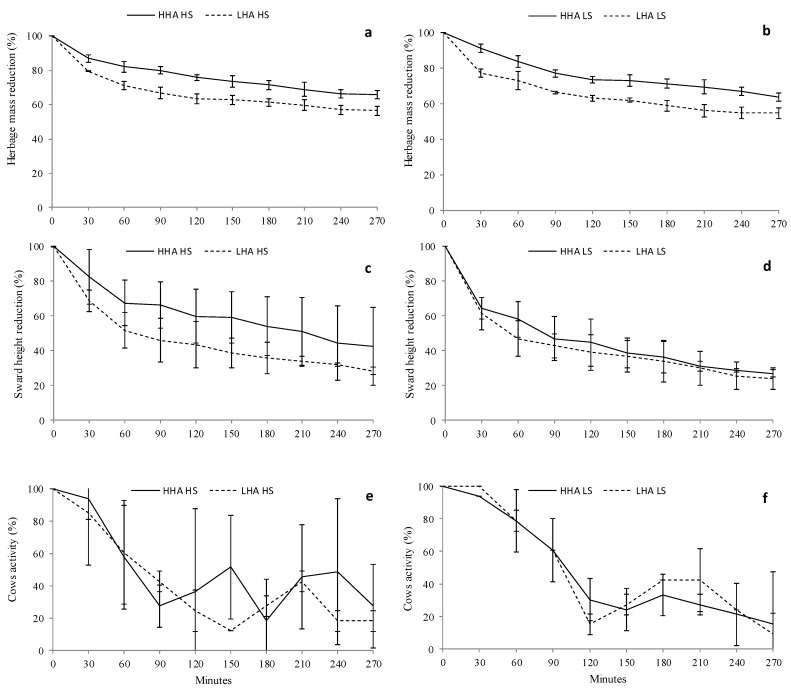
Herbage-mass decline (**a**,**b**), sward height decline (**c**,**d**) and proportion of cows engaged in grazing activity (**e**,**f**) during grazing sessions of 270 min. Data are means drawn from three measurement periods. Curves were fitted to high (solid line) and low (dashed line) daily herbage-allowance treatments. Figures on the left and right are high (HS) and low (LS) levels of maize silage supplementation values, respectively. Vertical bars represent s.e.m. values.

The herbage mass and USSH of pasture showed an exponential reduction as the grazing session progressed (Figure 2). Herbage mass reduction was faster (*p* < 0.001) under the low DHA regime compared with the high DHA (Figure 2a,b). The total HM reduction reached 44.2% and 35.2% under low and high DHA conditions, respectively. However, the average USSH reduction was unaffected by DHA and reached 65% under high DHA and 74% under low DHA conditions (Figure 2c,d). Neither the level of MSS nor the interaction between DHA and MSS level had any effect on the herbage depletion (herbage mass and sward height).

Cow grazing activity was unaffected by DHA (*p* = 0.122, Figure 2e,f) or MSS (*p* = 0.261). No interaction between these main factors was recorded (*p* = 0.247). However, the time had a significant effect on the grazing activity (*p* < 0.001) and the dairy cows were more active at the beginning of the grazing session. In fact, during the first 40 min of the grazing session, more than 90% of the cows were grazing, while the grazing activity was reduced, on average, to 17.4% after 270 min from the start of the grazing session under high and low DHA conditions.

**Figure 3:**
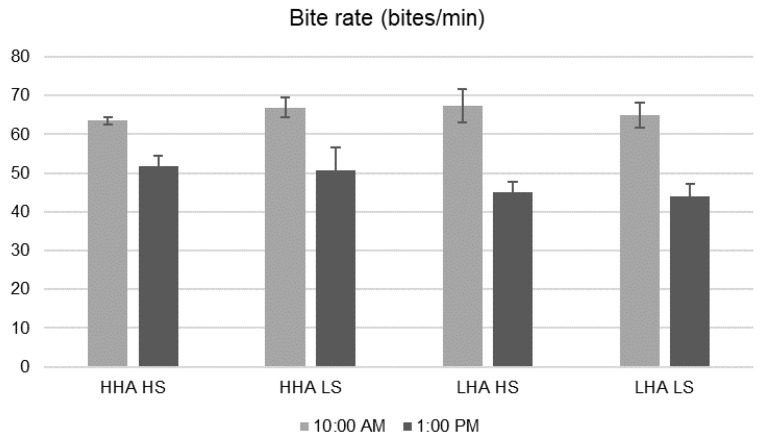
Bite rate of grazing dairy cows during grazing sessions of 270 min. HHA: high herbage allowance (25 kg DM/cow.day). LHA: low herbage allowance (17 kg DM/cow.day). HS: high silage supplementation (9 kg DM/cow.day). LS: low silage supplementation (9 kg DM/cow.day). Vertical bars represent s.e.m. values.

There was an interaction effect between DHA and the time (start vs. end, *p* = 0.001) on the biting rate (Figure 3). The biting rate was similar between cows under low and high DHA at the middle of the grazing session (average 66.0 bites/min at 10:00 a.m.), but it was greater in the cows grazing under high DHA compared with low DHA (51.6 vs. 44.4 bites/min) at the end of the grazing session (13:00).

Differences in the proportions of the morphological components that were observed during the grazing session were not affected by the DHA level (*p* > 0.05, Table 4). The proportion of lamina decreased as the grazing session progressed while the proportions of sheath and dead material increased. Overall, after 120 min of grazing, the proportion of lamina decreased from 89.5% to 76.3% while the proportion of sheath and dead material increased from 2.9% and 7.6% to 7.6% and 17.6%, respectively (average values between both DHA treatments).

### 3.5. Pasture Regrowth Rate

The sward leaf-production levels were not affected by the DHA regimes, averaging 14.2 days/leaf (Table 5). Offering low DHA levels decreased the tiller production rate by 4.8 days/tiller and the daily increase of HM by 8.2 kg DM/ha compared to the high DHA regime (Table 5). The highest lamina growth was registered in the high DHA treatment (+0.16 cm/day). Neither DHA nor the level of MSS had an effect on any of the pasture-regrowth variables evaluated.

### 3.6. Animal Measurements 

Cows grazing in low DHA pastures reduced individual milk production by 1.19 kg/day and milk protein from 3.34% to 3.26% when compared to the high DHA cows (Table 6). The milk fat concentration increased at a low DHA from 3.87% to 4.13% compared to the high DHA values. Neither the MSS level nor the interaction between DHA and the MSS level had any effect on the milk yield and composition.

## 4. Discussion

### 4.1. Grazing Management

In this study, all the cows grazed in similar pre-grazing pasture conditions. The average values recorded for pre-grazing HM and pre-grazing CSH (2292 kg DM/ha and 8.1 cm, respectively) agree with those reported by Ruiz-Albarrán et al. [22] and King and Stockdale [42] for cows grazing in an autumn pasture and fell within the range of recommended pre-grazing HM in Southern Chile during the autumn [43]. Although it is widely accepted that an increase in pasture utilization can be achieved by decreasing DHA [44] or increasing the stocking rate [45], the total amount of herbage utilized is seldom reported. Average pasture utilized per grazing event was 825 kg DM/ha and 784 kg DM/ha for low and high DHA, respectively. Given that cows offered a low DHA had approximately a 29.4% less offered area (−32.4 m^2^/cow.day) than those on the high DHA regime, the post-grazing HM and post-grazing CSH values decreased by 7.5% and 10.8%, respectively. In addition, the treatment under the low DHA conditions exhibited an increased instantaneous stocking rate (+1.1 cows/ha/day) compared to those of the high DHA condition, whereas the efficiency of harvesting remained unaffected. Despite these differences in the post-grazing residual, no differences were observed in the apparent forage DMI per hectare between DHA treatments. Therefore, the apparent forage DMI per cow increased on average by 0.28 kg DM per kg of increment in DHA. This agrees with the range reported previously of 0.10 to 0.30 kg DM when the DHA is estimated above the ground level [6,10,46,47,48]. The increased forage DMI per cow in the high DHA treatments might be explained by the greater amount of forage allocated per cow [49] and the greater grazing area.

### 4.2. Sward Characteristics

Given that the experiment was carried out in autumn and early winter with pastures dominated by *L. perenne* L. in the vegetative stage, a high proportion of leaves and high nutritive quality of the forage offered (>4 cm of height) were found. The forage CP and ME concentrations in available pasture found in this study fall within the range of high-quality pastures from Southern Chile in the autumn [50]. Regarding the distribution of HM in the vertical plane, the greater proportion of HM was positioned below 4 cm in height, which averaged 53.1% DM between both DHA levels, which is in agreement with the study carried out in Valdivia (Southern Chile) during a four-year period [15].

The absence of differences between the DHA levels in botanical and chemical compositions, pasture density, and plant weight could be explained by the short experimental period used and by the fact that all the pastures used in the present study had similar characteristics, according to the measurements carried out during the pre-experimental period. Moreover, the pre-grazing HM was controlled for each of the four herds and the post-grazing sward height corresponded to the range of 4 to 6 cm, as recommended by Donaghy and Fulkerson [51] for an effective grazing management system for ryegrass-based pastures. The lack of short-term DHA effects on all sward characteristics evaluated in the present study are consistent with the long-term effect reported by Merino et al. [15] when comparing DHA of 20 kg DM/cow.day and 30 kg DM/cow.day in the spring. Coincidently, in a recent study performed in Uruguay studying the effect of DHA (high, 38.4 kg DM/cow.day, medium, 30.3 kg DM/cow.day and low, 26.8 kg DM/cow.day, measured above the ground level) on botanical composition of the sward for one year, they found no effect of DHA on a pasture comprising *Festuca arundinacea*, *Trifolium repens*, and *Lotus corniculatus* in any season [52].

### 4.3. Herbage Depletion and Changes in Morphological Components during Grazing

Herbage depletion showed an exponential reduction as the grazing session progressed. Cows consumed close to 31% of the HM and reduced approximately 55% of the USSH with respect to the corresponding initial value during the first 120 min of the grazing session. The amount of pre-grazing HM available to be eaten (above 4 cm) at the start of the grazing session was 1198 kg DM/ha and 1286 kg DM/ha for low and high DHA, respectively, from which the amount of lamina was on average 1112 kg DM/ha for the low DHA and 1160 kg DM/ha for high DHA. Cows grazing under the low DHA conditions consumed the pasture upon a faster offer than those under the high DHA conditions as a consequence of the lower herbage availability and the greater grazing pressure. However, the USSH reduction rate was unaffected by the DHA conditions, likely as a consequence of the absence of differences when the cows were fed with 4.5 kg DM/cow.day of maize silage and because of the similar pre-grazing pasture conditions in terms of the vertical distribution of HM.

As the sward was grazed down, the proportion of lamina was reduced while the proportion of stem and dead material increased, which coincided with the findings of McGilloway et al. [53]. Thus, after 120 min, the reduction in the amount of available leaf-lamina (>4 cm) was, on average, of 459 kg DM/ha whereas dead material was increased by 61 kg DM/ha, respectively. Despite the fact that the dynamics of change throughout the grazing session observed in all proportions of sward morphological components in the pasture on offer (>4 cm) did not vary between DHA treatments, the amount of leaf-lamina present after 120 min from the star of the grazing session was 106 kg DM/cow.day lower at the low DHA when compared with high DHA treatments.

Cows with a low DHA and a low level of MSS achieved greater bite rates at the beginning of the grazing session than those under a high DHA condition, likely because the motivation to graze was greater compared with the other feeding regimes and because the substitution of pasture by supplements decreases as the DHA decreases [11]. The lower bite rate at the end of the grazing session in cows grazing on low DHA is likely attributable to a decreased motivation to continue grazing, which is caused by a higher sward bulk density [54] and a lower proportion of green lamina [55] in the lower strata compared with the upper strata. Moreover, the lower bite rate and grazing activity observed near the end of the grazing session can also be explained by a higher sward bulk density [54] and a lower amount of green lamina [55] available to be eaten. While the total HM was reduced by 1187 and 924 kg DM/ha under low and high DHA condition, respectively, the reduction in the USSH reach 12 cm on average without differences between DHA. Regarding the total available green-lamina reduction, this reached 571 and 579 kg DM/ha under low and high DHA conditions, respectively. The sward height [56] and the proportion of green lamina in the deeper strata [57] are the most important structural sward variables that affect the short-term herbage intake rate. In addition, it is largely accepted that dairy cows are able to adapt their grazing behaviour, according to the grazing conditions, and have the ability to anticipate access to a new strip after milking, as described by Peyraud et al. [6]. Accordingly, the restrictive herbage availability and the relatively high supplementation level (from 4.5 to 9 kg DM/cow.day) would also have decreased the intake rate.

### 4.4. Pasture Regrowth

Net production of HM represents the balance between pasture growth, senescence, and tiller population density [35]. Pasture growth is a function of the leaf growth and leaf production as well as the tiller (or stolon) production rates [58]. Moreover, the lamina growth rate is linearly related to the mean daily temperature [16]. In turn, leaf and tiller production are continuous processes regulated by environmental conditions (temperature, luminosity) and pasture management (especially nitrogen fertilization). The results of our study showed that DHA did not affect the leaf production rate. That likely was because the pre-grazing HM did not differ between DHA treatments and because this process depends mostly on the temperature [59]. On the other hand, the greater tiller production rate at a high DHA could be the result of the grazing management that resulted in a greater amount of water-soluble carbohydrates available in the stubble for regrowth [60].

The pasture growth rates decreased progressively as the autumn season progressed and was 28% greater in the high DHA than in the low DHA conditions. It can be attributed to the greater photosynthetically active area remaining in the post-grazing residue [52]. The mean pasture growth rates agreed with the values that were expected in the permanent pastures of Southern Chile during the autumn, which were in the range of 30 to 50 kg DM/day [16].

### 4.5. Animal Performance and Grazing Behaviour

As part of two parallel studies conducted on the same herd and paddock with a focus on nutritional aspects [61] and energy metabolic response [62,63], the intake of MSS and concentrate was measured. Cows, in total, consumed 12.1 kg DM forage per day (pasture plus MSS) and 3 kg of concentrate, with no difference among groups.

The greater pasture intake obtained at the high DHA (representing 54.3% of the total DMI vs. 39.7% at the low DHA) was sufficient to increase the individual milk production, which increased by 0.15 kg per kg of increased DHA. This result was associated with a decreased grazing severity [64] and, consequently, the ability of the cow to select greater quality herbage within the sward was greater compared to cows grazing at a low DHA [65]. Moreover, it can be assumed that the increase in herbage DM intake when cows grazed on the high DHA condition could increase the energy intake and decrease the extent of the negative energy balance commonly found in the first months of lactation, and, thus, improve milk production [66]. This assumption could be supported by the results reported by Morales et al. [63] who indicated that increasing DHA from 17 to 25 kg DM per cow decreases *B*-hydroxybutyrate in plasma from 1.12 to 0.91 mmol L^−1^.

The increased milk production at the high DHA shown in our study was similar to the findings of Pulido et al. [48] and Pérez-Prieto et al. [44], who reported that individual milk production per cow increased by 0.12 and 0.09 kg per kg of DHA offered, respectively. Greater responses were observed by Wales et al. [67], who reported that the milk yield increased by 0.99 kg per kg of DHA increase. In contrast, Ruiz-Albarrán et al. [22] and Kennedy et al. [68] did not find evidence of the effect of DHA when 17 and 25 kg DM/cow.day (measured above ground level) and when 13, 16, and 19 kg DM/cow.day (>4 cm height) were used, respectively.

Experiments using the stocking rate (SR) as the main factor governing milk output per cow and per unit pasture area have shown that, as the SR increases, the individual milk production is reduced because DHA, and, consequently, the offered area and forage DMI are reduced [49]. Despite the individual milk production decreased by 1.2 kg/day (92.4 kg over a 77-day period) at a low DHA, the results of this study showed that, with a 1 cow/ha increase in SR, the milk output per hectare increased by 24.3% (+1510 kg). These results are consistent with the long-term effect of DHA on milk production, as reported by Merino et al. [15], who found that, with a 1 cow/ha increase in SR, the milk output per hectare increases by 27.3%.

The milk fat concentration decreased by 0.33 g/kg per kg of increase in DHA. The lower milk fat concentration observed in this study was a consequence of the greater individual milk production when cows grazed at a high DHA. This effect was relatively greater than those of Pérez-Prieto et al. [44] in an experiment carried out in the winter using DHA between 19 to 46 kg OM/cow.day. The absence of an interaction effect between DHA and the level of MSS on milk production and milk fat concentration observed in this study has been previously described by Delaby et al. [69], who applied a DHA ranging from 12 to 22 kg DM/cow.day.

The CP supply from high-quality pastures in the autumn usually exceeds requirements for milk production of dairy cows, whereas the energy intake is the main limiting nutrient [70]. Therefore, the use of supplementary feed is necessary to ensure a stable supply in quantity and quality of energy to avoid the energetic costs associated with excreting N through urea synthesis [71] and to optimise rumen microbial synthesis, which improves dietary N utilization (milk N in relation to N intake) [72].

The higher milk protein observed in our study at the high DHA was not related to the quality of the feed provided (because forage and MSS quality did not differ in CP nor ME concentrations) or to the total DM intake. The increase in milk protein content when more herbage was offered could be explained by the decrease in the plasma concentration of *B*-hydroxybutyrate reported by Morales et al. [63], which represents a positive effect on the energy metabolism. The milk protein concentration increased by 0.01 g/kg per kg of increase in DHA, which is similar to the results reported by Delaby et al. [69].

### 4.6. Final Remarks

In a high-quality pasture, allocating herbage at 17 kg DM/cow.day with 4.5 kg/cow.day MSS was appropriate for improving both herbage utilization and milk production per hectare while maintaining the short-term conditions of a pasture grazed by dairy cows in the autumn. This would represent an economical benefit, as long as the potential marginal increase in costs does not counteract the marginal increase in the revenues from the additional milk produced, compared with offering more grazing area or a greater amount of supplement to animals, as well as a strategy to enhance the sustainability, both economic and biological, of pasture-based dairy production systems. However, the beneficial effects found in this research are not guaranteed when the overall management does not satisfy the cow’s nutrient requirements. Therefore, the health status and welfare of lactating dairy cows must be monitored. Moreover, the level of DHA must be adjusted in the following spring and summer periods.

## 5. Conclusions

To improve the profitability of pasture-based dairy systems, daily herbage allowances should improve the efficiency of pasture utilization and milk output per hectare. This study shows that, over the short-term, decreasing the daily herbage allowance by 32% (from 25 to 17 kg DM/cow.day) reduced milk production levels of autumn calving dairy cows by 5.2% (−1.2 kg/day), while increasing the milk output per hectare by 24.3%. In absolute values, the variation of milk output per ha is, thus, 16 times greater than the reduction of individual milk production that was reached while maintaining the quality of high-quality pasture grazed by dairy cows. Supplementation with 4.5 kg/cow.day of maize silage was sufficient to maintain the milk yield and composition per cow and to improve the productive response per hectare, without affecting the pasture condition.

## Figures and Tables

**Table 1:** Effects of daily herbage allowances (DHA) and maize silage supplementation (MSS) level on pre-grazing and post-grazing herbage mass, pre-grazing and post-grazing compressed sward height, herbage dry matter (DM )intake, offered area, and the efficiency of harvesting estimated daily using a rising plate-meter.

Variable	DHA (kg DM/cow.day)		MSS (kg DM/ cow.day)		SEM	Significance		
	17	25	4.5	9		DHA	MSS	DHA × MSS
Herbage mass (kg DM/ha)								
Pre-grazing	2255	2330	2298	2287	91.9	n.s.	n.s.	n.s.
Post-grazing	1430	1546	1455	1521	24.2	<0.05	n.s.	n.s.
Compressed sward height (cm)								
Pre-grazing	7.9	8.2	8.1	8.1	0.4	n.s.	n.s.	n.s.
Post-grazing	4.4	5.0	4.6	4.8	0.1	<0.05	n.s.	n.s.
Apparent pasture intake								
Intake per hectare	825	784	843	776	70.8	n.s.	n.s.	n.s.
Intake per cow (kg DM/cow.day)	6.0	8.2	7.4	6.8	0.5	<0.01	n.s.	n.s.
Offered area (m^2^/ cow.day)	77.9	110.3	93.7	94.5	5.0	<0.001	n.s.	n.s.
Efficiency of harvesting (%)	35.2	32.6	35.5	32.5	1.7	n.s.	n.s.	n.s.

n.s.: not significant (*p* > 0.05).

**Table 2:** Effect of daily herbage allowance (DHA) on the vertical distribution of dry matter (DM) herbage mass from the top of the canopy to the ground level.

Canopy Level	DHA (kg DM/cow.day)		SEM	Significance
	17	25		
>20 cm	2.0	4.8	0.9	0.079
16–20 cm	4.1	5.7	0.7	0.082
12–16 cm	7.0	9.2	0.7	<0.05
8–12 cm	11.8	12.5	0.8	n.s.
4–8 cm	19.9	16.7	1.6	n.s.
<4 cm	55.2	51.1	3.6	n.s.

Vertical distribution of herbage mass is expressed as a percentage of DM. n.s.: not significant (*p* > 0.05).

**Table 3:** Effect of daily herbage allowance (DHA) and maize silage supplementation (MSS) level on chemical and botanical composition, pasture density, and plant weight.

Variable	DHA		MSS		SEM	Significance		
	(kg DM/cow.day)		(kg DM/cow.day)					
	17	25	4.5	9		DHA	MSS	DHA × MSS
Chemical composition								
DM (% of DM)	12.6	12.7	12.7	12.7	0.2	n.s.	n.s.	n.s.
CP (% of DM)	24.9	24.9	24.6	25.2	0.2	n.s.	n.s.	n.s.
NDF (% of DM)	48.4	48.7	48.6	48.6	0.3	n.s.	n.s.	n.s.
Ash (% of DM)	9.5	9.8	9.6	9.7	0.1	n.s.	n.s.	n.s.
ME (MJ /kg DM)	11.7	11.7	11.7	11.7	1.9	n.s.	n.s.	n.s.
Botanical composition (% of DM)								
*Lolium perenne*	93.9	91.33	89.7	93.1	3.1	n.s.	n.s.	n.s.
*Agrostis capillaris*	0.1	1.4	2.3	1.8	0.7	n.s.	n.s.	n.s.
Other grasses	2.3	2.22	2.1	2.9	0.9	n.s.	n.s.	n.s.
*Trifolium repens*	2.1	3.88	2.0	1.7	1.3	n.s.	n.s.	n.s.
Broadleaf species	1.7	1.22	2.7	0.5	0.9	n.s.	n.s.	n.s.
Pasture density (tiller or number of plant per m^2^)								
Grass species	8089	8859	8339	8603	567.5	n.s.	n.s.	n.s.
*Trifolium repens*	161	124	191	94	85.0	n.s.	n.s.	n.s.
Broadleaf species	621	473	486	608	177	n.s.	n.s.	n.s.
Plant weight (mg per tiller or plant)								
Grass species	14.7	17.1	15.8	15.9	2.1	n.s.	n.s.	n.s.
*Trifolium repens*	7.8	9.6	8.9	8.4	0.1	n.s.	n.s.	n.s.
Broadleaf species	40.2	43.4	40.9	43.4	5.0	n.s.	n.s.	n.s.

CP: crude protein; NDF: neutral detergent fibre; ME: metabolizable energy; n.s.: not significant (*p* > 0.05).

**Table 4:** Effects of daily herbage allowances (DHA) and maize silage supplementation (MSS) level on variations in the sward morphological components above 4 cm during grazing.

Morphological Component	DHA		MSS		SEM	Significance		
	(kg DM/cow.day)		(kg DM/cow.day)					
	17	25	4.5	9		DHA	MSS	DHA × MSS
Lamina (% of DM)								
Initial	90.2	88.7	91.0	87.9	1.0	n.s.	n.s.	n.s.
Middle	73.4	79.1	73.6	78.9	2.1	n.s.	n.s.	n.s.
Final	69.6	73.8	71.7	71.6	2.2	n.s.	n.s.	n.s.
Sheath (% of DM)								
Initial	2.7	3.2	2.6	3.2	0.4	n.s.	n.s.	n.s.
Middle	5.1	7.3	7.4	5.0	0.6	n.s.	n.s.	n.s.
Final	8.7	6.3	7.8	7.3	0.9	n.s.	n.s.	n.s.
Dead material (% of DM)								
Initial	7.1	8.1	6.4	8.9	0.8	n.s.	n.s.	n.s.
Middle	21.5	13.6	19.0	16.1	1.9	n.s.	n.s.	n.s.
Final	21.7	19.9	20.5	21.1	1.7	n.s.	n.s.	n.s.

DM: dry matter; n.s.: not significant (*p* > 0.05).

**Table 5:** Effect of daily herbage allowance (DHA) and maize silage supplementation (MSS) level on pasture regrowth rates.

Variable	DHA		MSS		SEM	Significance		
	(kg DM/cow.day)		(kg DM/cow.day)					
	17	25	4.5	9		DHA	MSS	DHA × MSS
Leaf production rate (days/lamina)	15.14	13.23	14.74	13.64	1.33	n.s.	n.s.	n.s.
Tiller production rate (days/tiller)	23.86	19.11	20.60	22.34	0.76	<0.001	n.s.	n.s.
Lamina growth (cm/day)	0.34	0.50	0.40	0.43	0.02	<0.001	n.s.	n.s.
Pasture growth (kg DM/ha/day)	29.32	37.44	31.22	32.45	2.05	<0.001	n.s.	n.s.

n.s.: not significant (*p* > 0.05).

**Table 6:** Effects of daily herbage allowances (DHA) and level of maize silage supplementation (MSS) on milk production levels and milk compositions of autumn-calving dairy cows.

Variable	DHA		MSS		SEM	Significance		
	(kg of DM/cow.day)		(kg DM/cow.day)					
	17	25	4.5	9		DHA	MSS	DHA × MSS
Milk production (kg/day)	21.99	23.18	22.53	22.64	0.15	<0.001	n.s.	n.s.
Milk fat (%)	4.13	3.87	4.02	3.97	0.09	<0.01	n.s.	n.s.
Milk protein (%)	3.26	3.34	3.29	3.32	0.04	<0.05	n.s.	n.s.

n.s.: not significant (*p* > 0.05).
